# The design of a novel arthroscopy shaver

**DOI:** 10.1038/s41598-022-17674-2

**Published:** 2022-08-12

**Authors:** Xuelian Gu, Shiting Yuan, Pengju Xu, Shanshe Xiao, Wentao Liu, Weiguo Lai, Zhi Chen, Peng Liang, Gaiping Zhao

**Affiliations:** 1 School of Medical Instrument and Food Engineering, University of Shanghai for Science and Technology, No. 516 Jungong Road, Shanghai, 200093 People’s Republic of China; 2 ShangHai Ligatech Bioscience Co. Ltd, 508 Tianchen Road, Shanghai, 201712 People’s Republic of China; 3 Shanghai BJ-KMC Medical Technology Co., Ltd., Building #23,528 Ruiqing RD, Zhangjiang High-Tech Park East, Pudong, Shanghai, 201712 People’s Republic of China

**Keywords:** Biomedical engineering, Engineering, Materials science

## Abstract

Cases of arthroscopic surgery have increased over the past two decades, and arthroscopic shaver systems have become a commonly used orthopedic tool. Nevertheless, most shavers generally have problems such as the cutting edge is not sharp enough and easy to wear. This paper aims to discuss the structural characteristics of BJKMC’s (Bojin^◊^ Kinetic Medical) novel arthroscopic shaver, the double serrated blade. The product's design and verification process are outlined. BJKMC’s articular arthroscopy shaver has a “tube in a tube” structure, comprising a stainless steel outer sleeve and a rotating hollow inner tube. The outer sleeve and inner tube have corresponding suction and cutting windows, and there are serrated teeth on the inner and outer casing. To verify the design rationality, it was compared to Dyonics^◊^’s equivalent product, the Incisor^◊^ Plus Blade. The appearance, cutting tool hardness, metal pipe roughness, cutting tool wall thickness, tooth profile, and angle, overall structure, and the key dimensions were examined and compared. Compared with Dyonics^◊^’s Incisor^◊^ Plus Blade, BJKMC’s Double Serrated Blade had a smoother working surface, harder and thinner blade head. Therefore, BJKMC’s product may have satisfactory performance when it comes to surgery.

## Introduction

Joints in the human body are a form of indirect connection between bone and bone. They are a complex yet stable structure, and play an important role in our daily life. Some diseases alter the stress distribution within joints, resulting in limitations and loss of function^1^. With traditional orthopedic surgery, it was difficult to accurately treat micro-traumas, and the recovery period after treatment was long. Arthroscopic surgery is a minimally invasive surgery that requires only a small incision; causes less trauma and less scarring, and has a faster recovery time and fewer complications. As medical equipment has developed, minimally invasive surgical techniques have gradually become the routine procedure for orthopedic diagnosis and treatment. It was not long after arthroscopic surgery was first performed on a knee that it was formally used as a surgical technique by Kenji Takagi and Masaki Watanabe in Japan^2,3^. Arthroscopy and arthroplasty were the two most important orthopedic improvements^4^. Today, arthroscopic minimally invasive surgery is used to treat numerous diseases and injuries, including osteoarthritis, meniscus injuries, anterior and posterior cruciate ligament injuries, synovitis, intra-articular fractures, patellar subluxation, cartilage injuries, and loose bodies.

Cases of arthroscopic surgery have increased over the past two decades, and arthroscopic shaver systems have become a commonly used orthopedic tool. There are now a variety of methods available to surgeons, including cruciate ligament reconstruction, meniscus repair, osteochondral transplantation, hip arthroscopy, and facet arthroscopy, which can be selected according to the surgeon’s preference^1^. As arthroscopic surgery procedures were extended to more joints, doctors were able to explore synovial joints and treat patients surgically in ways previously unimaginable^5^. At the same time, additional instruments were developed. They often comprised a control unit, a handpiece with a powerful engine, and a resection tool. Resection tools allow simultaneous and continuous aspiration and debridement^6^.

Due to the complexity of arthroscopic surgery, multiple instruments are usually required. The basic surgical tools used in arthroscopic surgery include arthroscopes, probe scissors, basket punches, clamp punches, arthroscopy knives, meniscus blades and shavers, electrosurgical tools, lasers, radiofrequency instruments, and other miscellaneous instruments^7^.

Shavers are important instruments in surgery. The main working principles of the planer machines used for arthroscopic surgery are divided into two types. The first is to remove degenerated cartilaginous debris by suction, including loose bodies and floating articular cartilage, as well as to flush the joints with plenty of normal saline to remove lesions and inflammatory mediators within the joints. The other is to gouge and remove articular cartilage that has separated from the subchondral bone, and to repair defects in worn cartilage. The torn meniscus was excised and shaped into worn and broken meniscus. Shavers are also used to remove part or all of the inflammatory synovial tissue such as hyperplasia and thickening^1^.

**Figure 1:**
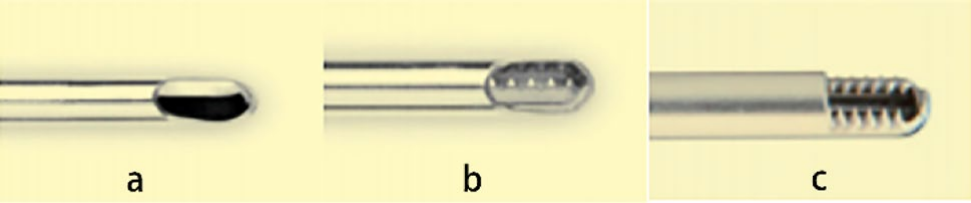
Types of arthroscopy shavers^9^.

Most minimally invasive surgical planer knives are designed to have a cutting part with a hollow outer sleeve and a hollow inner tube. They seldom have serrated teeth for the cutting edge^8^. Different cutting tips give the shaver different levels of cutting power. Common arthroscopy shaver teeth are divided into three categories (Fig. 1): (a) smooth inner and outer tube; (b) smooth outer tube and toothed inner tube; (c) toothed (can be razor edged) inner and outer tube^9^. Their sharpness to soft tissue was increasing. The average peak force and cutting efficiency of serrated teeth of the same specification are better than the flat ruler^10^.

Nevertheless, there are many problems with the currently available arthroscopic shavers. First, the cutting edge is not sharp enough, which can easily cause blockages when cutting soft tissue. Second, the shaver can only cut soft synovial tissue—doctors have to polish the bone using burrs. Therefore, frequent blade changes are required during surgery, increasing the operation time. Cutting damage and wear of the shaver are also widespread problems. Precision machining and precision control do lead to a unified evaluation index either.

The first problem is caused by the large clearance between the inner and outer blades and the lack of smooth working surface of the shaver. The solution to the second problem may be to increase the blade angle of the shaver and to increase the strength of the material in the design.

BJKMC’s novel double serrated blade-type arthroscopic shaver has the potential to solve the problems of blunted cutting edges, easy blockage, and fast tool wear. In order to verify the practicality of BJKMC’s novel shaver design, it was compared to Dyonics^◊^’s equivalent product, the Incisor^◊^ Plus Blade.

## Design and validation

### Design and specimen preparation

**Figure 2:**
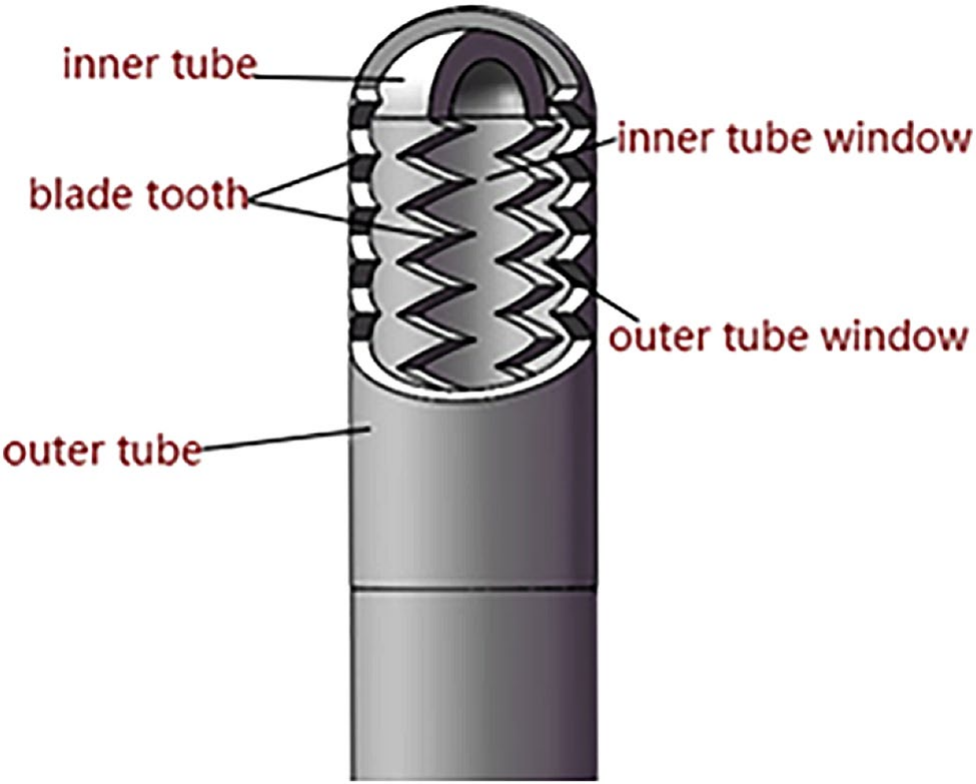
The Structural diagram of the shaver blade.

The novel articular arthroscopy shaver had a 'tube in a tube' structure, comprising a stainless steel outer sleeve and a rotating hollow inner tube, with corresponding suction and cutting windows on the outer sleeve and inner tube. There are serrated teeth on both the internal and external cannula. During operation, a power system drives the rotation of the internal tube and the tooth biting of the external tube in coordination with the cutting. The finished cutting tissues and loose bodies are extracted from the joint through the hollow internal tube. To improve the cutting performance and efficiency, a concave tooth structure was selected. Laser welding was used to synthesize parts. The general double serrated shaver head structure is shown in Fig. 2.

In terms of the overall structure, the external diameter of the front end of the arthroscope shaver was slightly smaller than that of the rear end. Because both the tip and the edge of the cutting window can scour and damage the articular surface, the shaver should not be forced into joint spaces. Besides, the width of the shaver's window should be large within a reasonable range. The wider the window, the stronger the organizational ability of the shaver in cutting and suction, and the better window blockages can be avoided.

**Figure 3:**
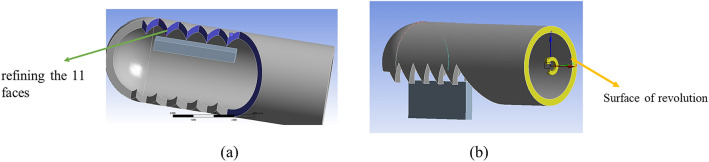
Mesh and boundary condition settings. (**a**) Mesh refinement. (**b**) Rotation face of shaver blade.

To explore the influence of tooth shape on cutting force. The 3D models of shaver were created using the SolidWorks software (SolidWorks 2016, SolidWorks Corp., MA, US). The outer sheath models with different tooth shapes were imported into the finite element software (ANSYS Workbench 16.0, ANSYS Inc., US) for mesh generation and stress analysis. The mechanical properties of the materials (elastic modulus and Poisson's ratio) are shown in Table 1. The mesh density used for soft tissue is 0.05 mm and we refine the 11 faces of the planer in contact with the soft tissue (Fig. 3a). The numbers of nodes in the entire model are 40,522 and the number of meshes are 45,449. In the boundary condition setting, we completely limit the 6 degrees of freedom given to the 4 sides of the soft tissue and the shaver blade rotates 20° around the X axis (Fig. 3b).

**Table 1:** Mechanical properties used for the FEA model^11–13^.

Material	Elastic modulus (GPa)	Poisson’s ratio
1RK91	190	0.31
Soft tissue	0.033	0.478

**Figure 4:**
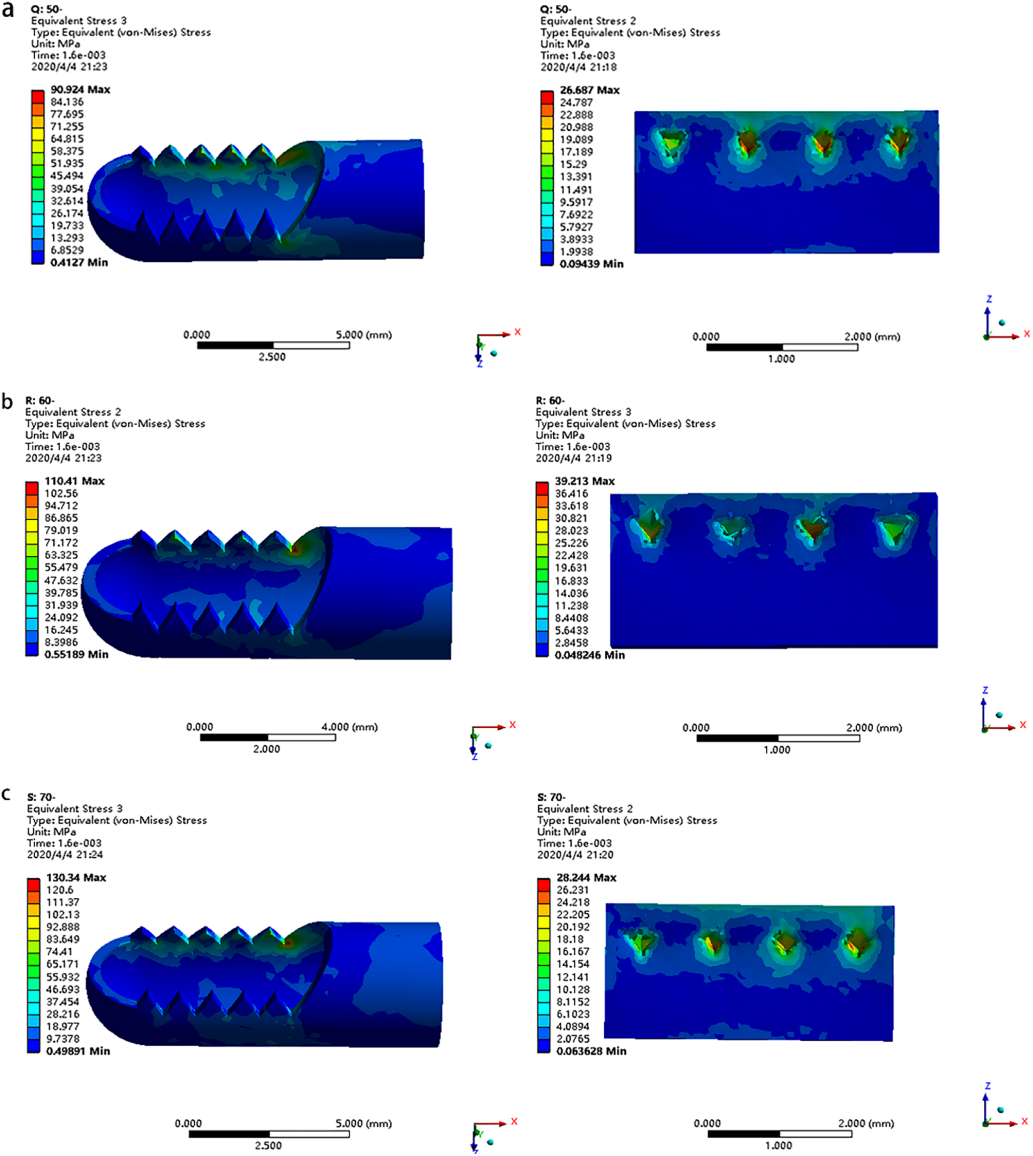
The stress distribution of the shaver and soft tissue when the outer sheath of the shaver with different tooth shapes is used to cut soft tissue: (**a**) 50° tooth profile, (**b**) 60° tooth profile, (**c**) 70° tooth profile.

From the analysis of three shaver models (Fig. 4), the maximum stress point appears at the structural mutation, which conforms to the mechanical properties. The shaver is disposable instrument^4^ and there is little risk of blade fracture under single use. Therefore, we mainly focus on its cutting performance. The maximum equivalent stress acting on soft tissue can reflect this property. Under the same working conditions, when the maximum equivalent stress is the largest, it is preliminary considered that its cutting performance is the best. From the stress on the soft tissue, the maximum shear stress on soft tissue was generated by the shaver with the tooth profile of 60° (39.213 MPa).

### The validation method

**Figure 5:**
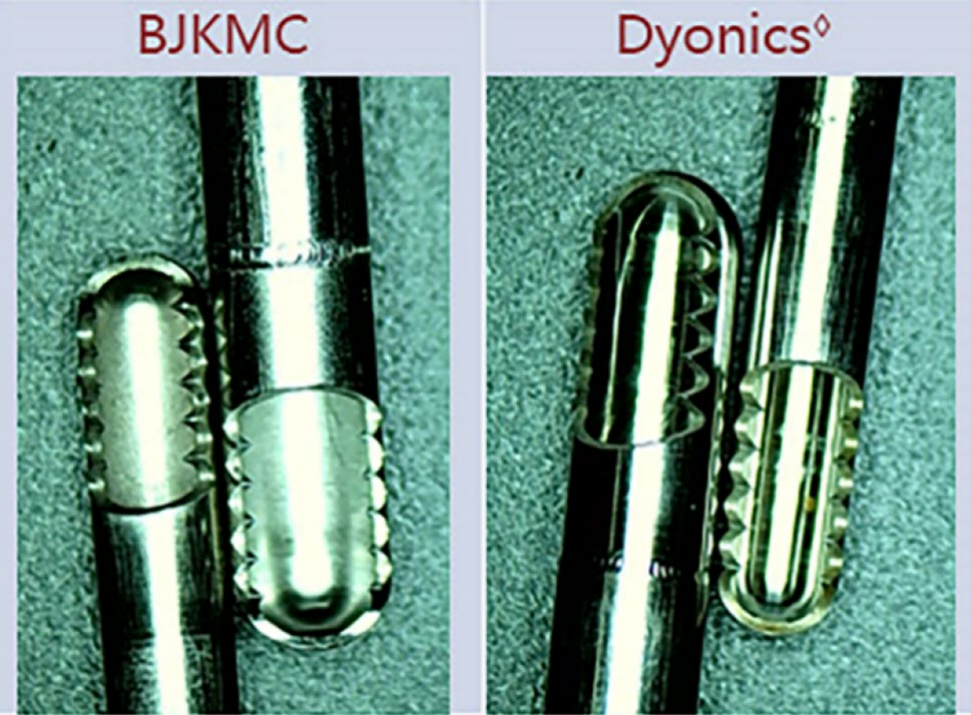
The double serrated blade (BJKMC) and the Incisor^◊^Plus Blade (Dyonics^◊^).

To verify the design rationality of BJKMC’s novel shaver blade, it was compared with Dyonics^◊^’s equivalent product, the Incisor^◊^ Plus Blade (Fig. 5), which has the same specifications. Three of the same type of each product were used in all experiments. All shavers used were new and without damage.

**Figure 6:**
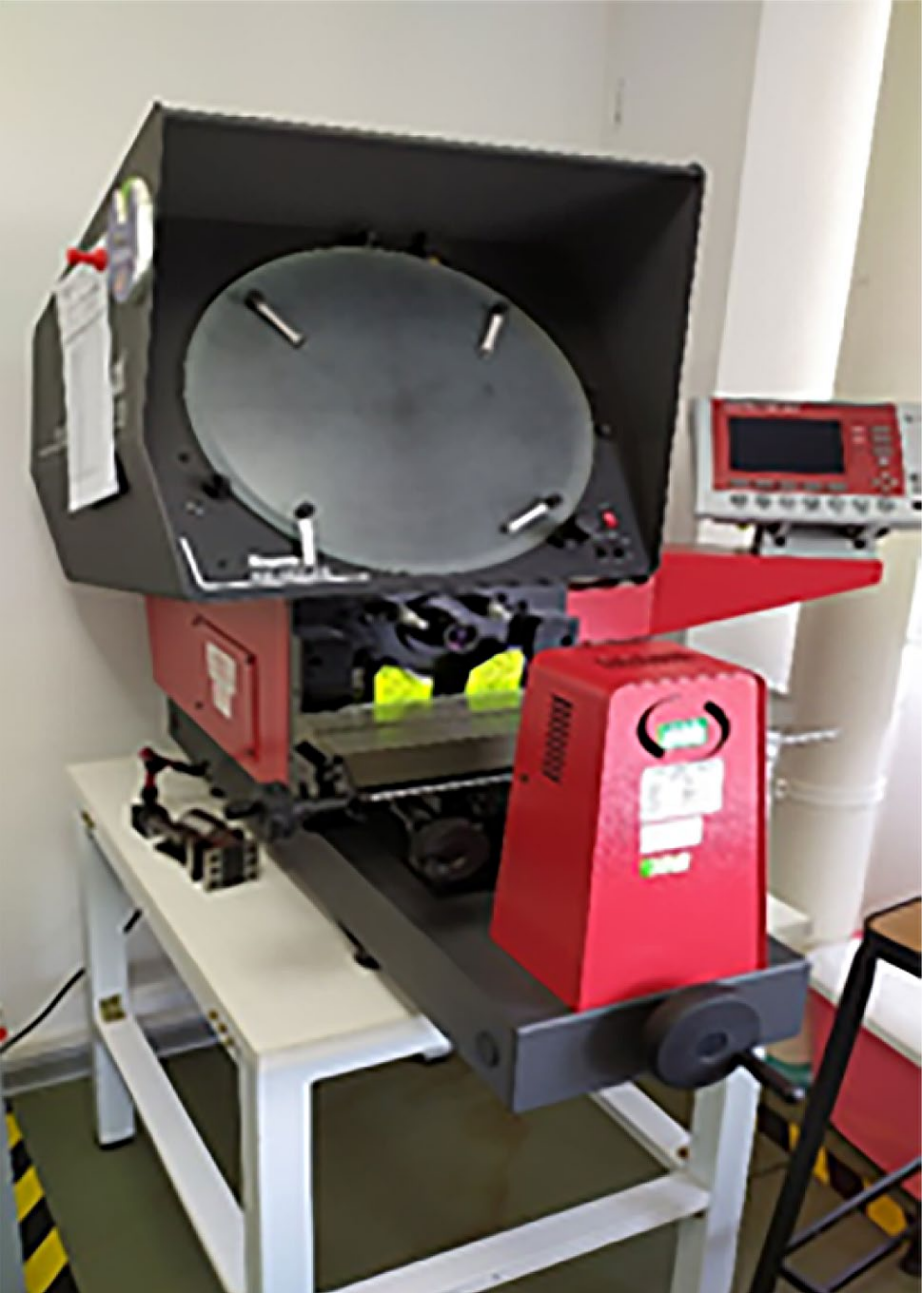
The profile projector (Starrett 400 SERIES).

Factors affecting the shaver performance include the hardness and thickness of the blade, the roughness of the metal tube, and the tooth profile and angle. In order to measure the profile and angle of the teeth, a profile projector (Starrett 400 SERIES Fig. 6) with a resolution of 0.001 mm was selected. In the experiment, the shaver head was placed on a worktable. The tooth profile and angle were measured according to the crosshair on the projection screen, and the measured value was determined using a micrometer as the difference between the two lines. The actual size of the tooth profile was obtained by dividing it by the magnification factor of the selected objective lens. In order to measure the tooth angle, fixed points on either side of the measured angle were aligned with the intersection of a sub-line on the shadow screen, and the reading was performed using the angle cursor of the worktable.

By repeating this experiment, the working length (inner and outer tube), the outer diameter of the front and back ends, window length and width, tooth height, and other key dimensions were measured.

**Figure 7:**
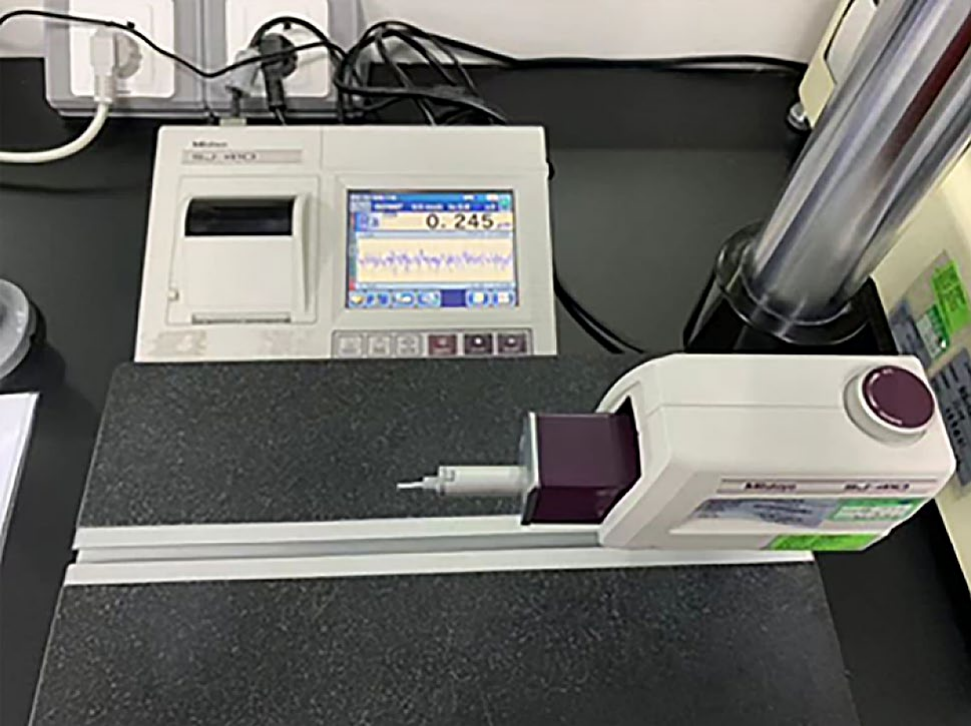
The point needle instrument.

The point needle instrument was used to test the surface roughness. The tip of the instrument moved horizontally across the specimen, perpendicular to the processing texture direction. The average roughness, Ra, was directly obtained from the equipment. Figure 7 shows the point needle instrument (Mitutoyo SJ-310).

The hardness of the shaver blades was measured according to ISO 6507-1:2005 Vickers hardness test^5^. The diamond indenter was pressed into the sample surface under a certain test force for a specified time. The diagonal length of the indentation was then measured after removing the indenter. The Vickers hardness is proportional to the quotient of the test force divided by the surface area of the indentation.

**Figure 8:**
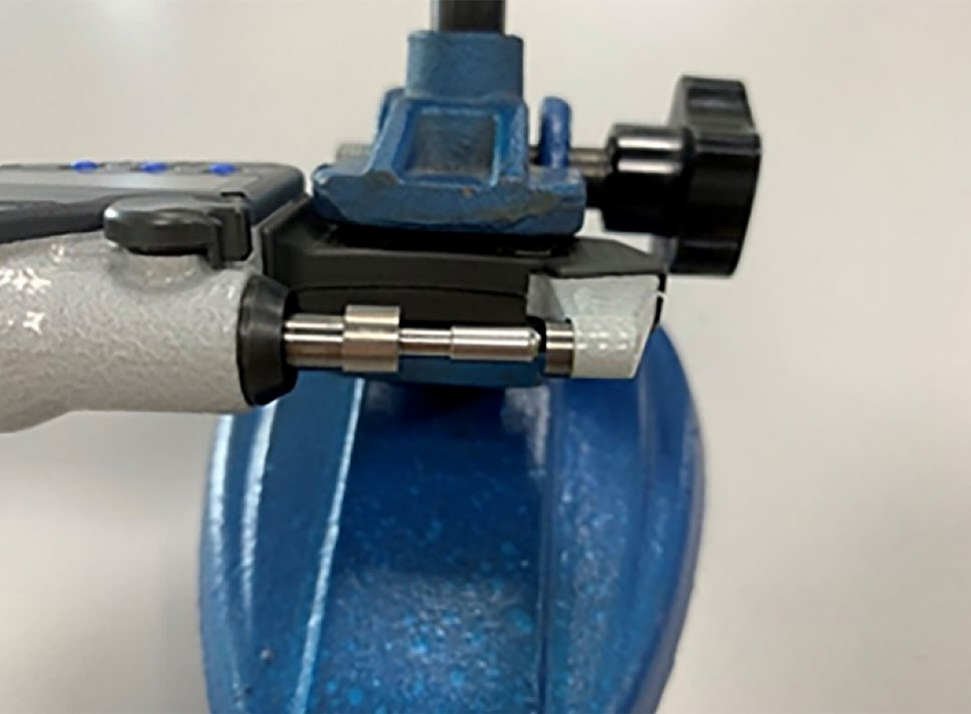
The experimental process of measuring the thickness.

The wall thickness of the shaver heads was measured gauge with an accuracy of 0.01 mm and measuring range of approximately 0–200 mm by inserting a cylindrical ball head. The wall thickness was determined as the difference between the outer and inner diameters of the tool. The experimental process for measuring the thickness is shown in Fig. 8.

The structural performance of BJKMC’s shaver was compared with that of Dyonics^◊^’s shaver of the same specification. The performance data of each part of the products were measured and compared. From the dimension data, the cutting ability of both products was predictable. Both products showed excellent structural performance; still conductive comparative analyses are required from various aspects.

### Consent for publication

All authors read and approved the final manuscript for publication.

## Results and discussion

### Tooth profile and angle

According to the angle experiment, the results are shown in Tables 2 and 3. There was no statistical difference between the mean values and standard deviations of the tooth profile angle data of the two products.

**Table 2:** Tooth profile and angle of BJKMC’s products.

Test project	Unit	BJKMC				
		1	2	3	AVE	SD
**Upper tooth profile**						
Out sheath	Degree	59.32	59.24	58.43	59.00	0.329
Inner knife		58.08	59.20	61.02	59.43	0.918
**Lower tooth profile**						
Out sheath	Degree	60.56	60.04	59.24	59.95	0.410
Inner knife		58.27	59.25	60.01	59.18	0.479

**Table 3:** Tooth profile and angle of Dyonics^◊^’s products.

Test project	Unit	Dyonics^◊^				
		1	2	3	AVE	SD
**Upper tooth profile**						
Out sheath	Degree	57.62	57.66	57.6	57.63	0.173
Inner knife		59.57	59.2	59.88	59.55	0.191
**Lower tooth profile**						
Out sheath	Degree	56.57	56.54	56.58	56.56	0.173
Inner knife		58.99	59.77	59.42	59.39	0.173

### Key dimensions of two kinds of shavers

**Figure 9:**
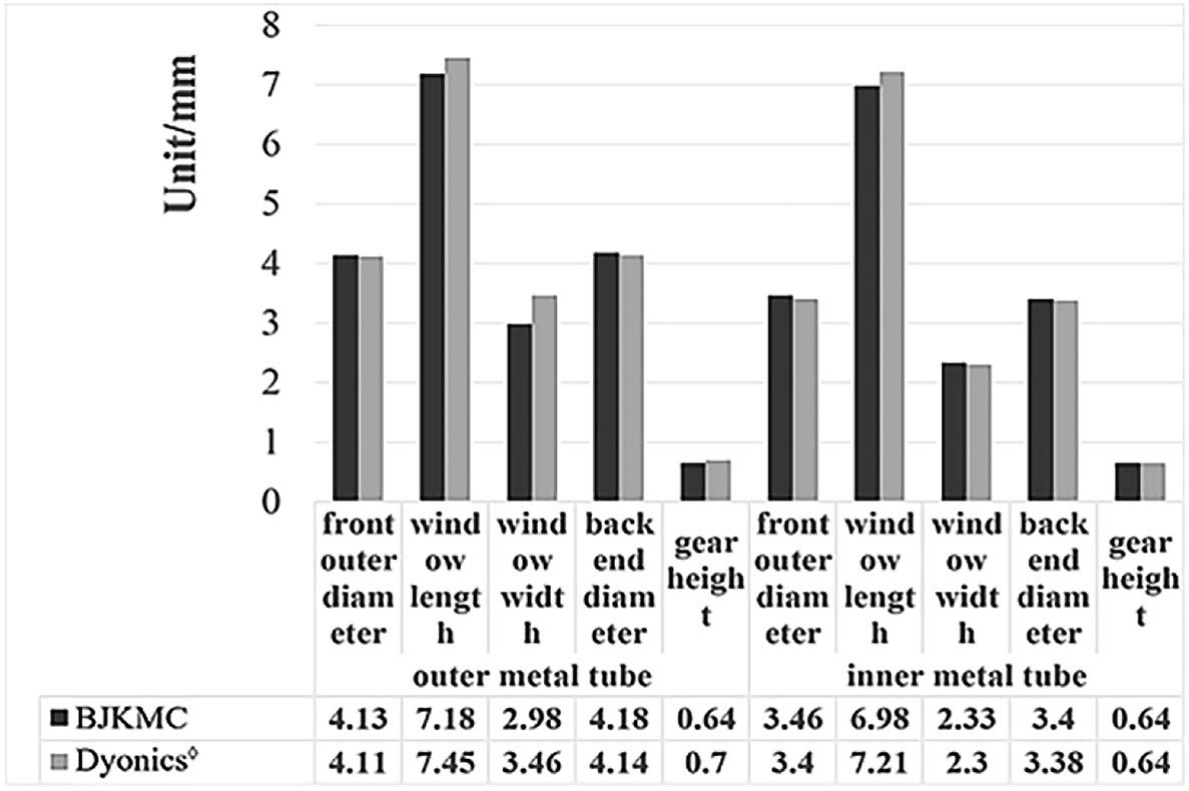
Key dimensions of two kinds of shavers.

Some key dimensions of the two products are compared in Fig. 9. In terms of the width and length of the inner and outer tubes, Dyonics’^◊^ Windows of the inner and outer tubes are little bit longer and wider than those of the BJKMC. This means Dyonics^◊^ may have more cutting space and the tube is not easily blocked. There was no statistical difference between the two products in other dimensions.

The parts of BJKMC’s shaver was joined by laser welding. Hence, there was no external pressure on the welding pieces. The welding parts are not affected by the thermal effect or thermal deformation. The welding site was narrow with a large penetration depth, and the welding part had high mechanical strength, strong vibration, and great impact resistance. Laser welded parts have high reliability during assembly^14,15^.

### Roughness of the tube

Surface roughness is a measurement of surface texture. It is considered to be the high-frequency and short-wavelength components of the measured surface, which determines the interaction between the object and the environment^16^. The inner surface of the outer sleeve and inner tube of the internal knife are the main working surfaces of the shaver. Reducing the roughness of the two surfaces can effectively reduce the wear of the shaver and improve its working performance.

**Figure 10:**
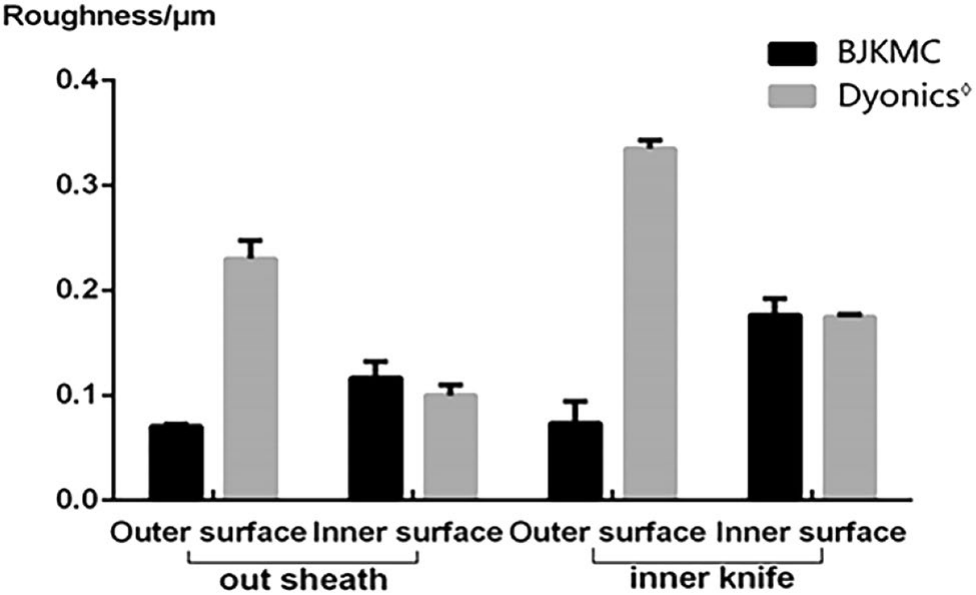
The data of roughness of metal tube.

The outer sheath surface roughness, inner and outer surface of the inner blade of the two kinds of metal pipes were obtained through experiments. Their average value is represented by Fig. 10. The inner surface of the outer sheath and the outer surface of the inner knife were the main working surfaces. BJKMC’s internal sheath surface and the outer surface of the internal knife had a lower roughness than those of Dyonics^◊^’s equivalent products (with the same specifications). This means that BJKMC's products may have satisfactory results in cutting performance.

### Hardness of blade

**Figure 11:**
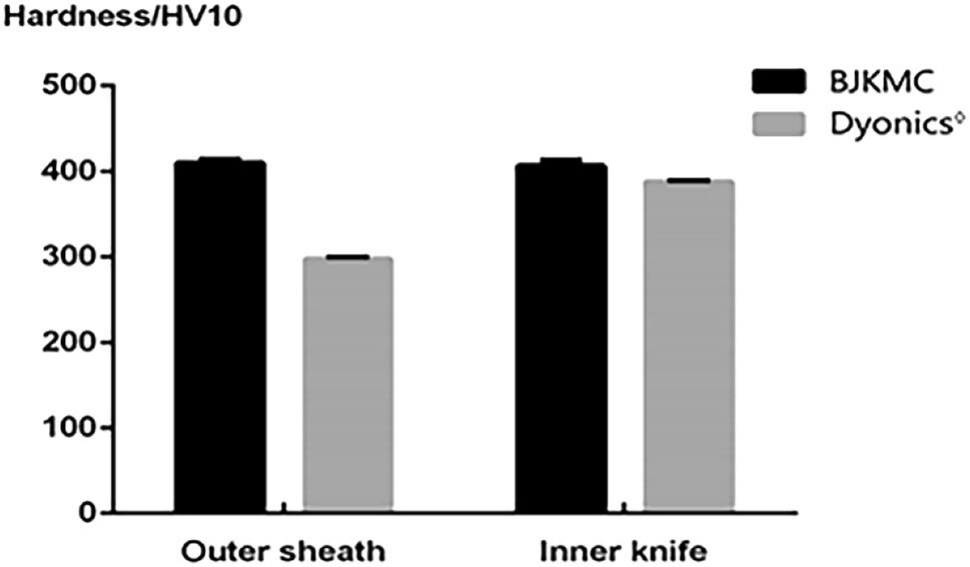
The data of hardness of shaver blade.

According to the blade hardness test, the experimental data of the two shaver blade groups are shown in Fig. 11. Since the shaver blade must have high strength, toughness, and plasticity, most arthroscope shavers are made of austenitic stainless steel. However, BJKMC’s shaver head is made of 1RK91 martensitic stainless steel. Martensitic stainless steel gives it higher strength and toughness than austenitic stainless steel^17^. The chemical elements in BJKMC’s product conformed to S46910 in the process of forging (ASTM-F899 surgical apparatus). This material has passed cytotoxicity tests and is widely used in medical apparatus.

Through the finite element analysis results, it can be seen that the stress concentration part of the shaver is mainly on the tooth profile. 1RK91 is a high-strength, high-toughness super martensite stainless steel, it can show good tensile strength at room temperature and high temperature. The tensile strength at room temperature can reach more than 2000 MPa, and the maximum stress value in the results of finite element analysis is about 130 MPa, which is far from the fracture limit of the material and we believe there is little risk of blade fracture.

### Thickness of shaver

The thickness of the blade directly affects the cutting performance of the shaver. The thinner the wall thickness, the better the cutting performance. BJKMC's novel shaver minimized the wall thickness of two rods rotating relative to each other, with a thinner knife head wall thickness than that of Dyonics^◊^’s equivalent product. The thinner knife can increase the cutting force of the blade head.

The data in Table 4 demonstrate that the wall thickness of BJKMC’s shaver obtained by the method of compression rotating wall thickness was thinner than the same specification of Dyonics^◊^’s shaver.

**Table 4:** The data of thickness of the blade.

Test project	Unit	BJKMC					Dyonics^**◊**^				
		1	2	3	AVE	SD	1	2	3	AVE	SD
**Thickness (blade)**											
Out sheath	mm	0.29	0.32	0.28	0.30	0.115	0.35	0.38	0.36	0.36333	0.002
Inner knife		0.33	0.33	0.34	0.33	0.006	0.37	0.38	0.38	0.37666	0.002

## Conclusion

According to the data of the comparative experiment, BJKMC’s novel arthroscopy shaver has no obvious structural differences from Dyonics^◊^’s equivalent model. Compared with Dyonics^◊^’s Incisor^◊^ Plus Blade in material properties, BJKMC’s Double Serrated Blade had a smoother working surface, harder and thinner blade head. Therefore, BJKMC’s product may have satisfactory performance when it comes to surgery. This study was on the prospective design, and the specific working performance should be verified in subsequent experiments.

## Data Availability

All the data are available within the manuscript.
